# Microemboli versus hypoperfusion as an etiology of acute ischemic stroke in Egyptian patients with watershed zone infarction

**DOI:** 10.1186/s41983-018-0045-8

**Published:** 2019-01-06

**Authors:** Ahmed ElSadek, Ahmed Gaber, Hossam Afifi, Sherin Farag, Nouran Salaheldien

**Affiliations:** Ainshams University, P.O. 11681, Elrehab City, Cairo Egypt

**Keywords:** Microemboli, Hypoperfusion, Watershed zone infarction

## Abstract

**Background:**

Brain perfusion is most likely to be impaired in border zone regions, and clearance of emboli will be most impaired in these regions of least blood flow. Severe occlusive disease of the internal carotid artery causes both embolization and decreased perfusion as well as some cardiac diseases that cause microembolization.

**Objectives:**

To differentiate between hypoperfusion and microemboli as etiology of acute ischemic stroke in watershed zone.

**Subject and methods:**

Fifty patients of acute ischemic stroke in watershed zones were recruited within 7 days from stroke onset. Methods used were transcranial Doppler (TCD) monitoring for the intracranial vessels to detect microembolic signals and magnetic resonance imaging (MRI) perfusion image to detect hypoperfusion signs.

**Results:**

We detect embolic causes of watershed infarction (WSI) by using TCD with 61.1% sensitivity and 84.4% specificity and hypoperfusion causes of WSI by using MRI perfusion studies with 94.9% sensitivity and 54.5% specificity.

**Conclusion:**

We detected the etiology of WSI, either embolic by using TCD or hypoperfusion by using MRI perfusion. The embolic causes of WSI usually cause external or mixed WSI, and hypoperfusion causes of WSI cause internal WSI.

## Introduction

Watershed strokes are named because they affect the distal watershed areas of the brain. The term “watershed” refers to those areas of the brain that receive dual blood supply from the branching ends of two large arteries [1]. These events are localized to two primary regions of the brain: Cortical watershed strokes (CWS), or outer brain infarcts, are located between the cortical territories of the anterior cerebral artery (ACA), middle cerebral artery (MCA), and posterior cerebral artery (PCA). Internal watershed strokes (IWS), or sub cortical brain infarcts, are located in the white matter along and slightly above the lateral ventricle, between the superficial systems of the MCA and ACA, or between the deep and the superficial arterial systems of the MCA [10]. The conventional theory implicates hemodynamic compromise produced by repeated episodes of hypotension in the presence of a severe arterial stenosis or occlusion. The lower perfusion pressure found within the border zone areas in this setting confers an increased susceptibility to ischemia, which can lead to infarction [5]. Radiologic studies also support the hypothesis that border zone infarcts distal to internal carotid artery disease are more likely to occur in the presence of a non-competent circle of Willis [9]. Hypoperfusion, or decreased blood flow, is likely to impede the clearance (washout) of emboli. Because perfusion is most likely to be impaired in border zone regions, clearance of emboli will be most impaired in these regions of least blood flow. Severe occlusive disease of the internal carotid artery causes both embolization and decreased perfusion. Similarly, cardiac disease is often associated with microembolization from the heart and aorta with periods of diminished systemic and brain perfusion [2]. Internal watershed infarcts are caused mainly by arterial stenosis or hemodynamic compromise. The greater vulnerability of internal border zones to hemodynamic compromise has been explained on the basis of anatomic characteristics of the cerebral arterioles within these zones [7]. The mechanism of external watershed infarction has been widely debated. Some studies have documented hemodynamic abnormalities as a mechanism of external watershed infarction especially in the anterior watershed zones. However, other studies revealed absence of evidence of hemodynamic impairment in external watershed infarction [3].

### Aim of the study

To differentiate between hypoperfusion and microemboli as etiology of acute ischemic stroke in watershed zone, with correlation between those etiologies and site of watershed zone infarction.

### Patients and methods

The study included 50 patients of acute ischemic stroke in watershed zones. They were recruited to this cross-sectional study from stroke unit at Ain Shams University Hospitals, Cairo, Egypt, within 7 days from stroke onset (Table 1).

**Table 1:** Demographic data including risk factors of stroke

Variables	
Age, mean (± SD)	62.78 (± 10.26)
Sex, *N* (%)	Male, 38 (76%)
	Female, 12 (24%)
Smoking, *N* (%)	Yes, 21 (42%)
	No, 29 (58%)
Diabetes mellitus (DM), *N* (%)	Yes (31%)
	No (19%)
Hypertension (HTN), *N* (%)	Yes (32%)
	No (18%)
Ischemic heart disease	Yes (14%)
	No (36%)
Dyslipidemic	Yes (0%)
	No (50%)
Atrial fibrillation	Yes (12%)
	No (38%)
Stroke/transient ischemic attack	Yes (12%)
	No (38%)

### Inclusion criteria and subject selection

Patients with acute watershed infarction (WSI) within 7 days from stroke in the age ranging from 37 to 81 years.

### Exclusion criteria

Transient ischemic attack; intracerebral hemorrhage; subarachnoid hemorrhage; lack of baseline data like transthoracic echo, carotid duplex, etc.; admission to the hospital more than 7 days from the onset of stroke; or refusal to participate in the study protocol.

### Ethical consideration

All patients or relatives were informed about the study and its possible benefits. A total of 50 patients of acute ischemic stroke in watershed zones within 7 days from stroke onset were enrolled. All patients had detailed neurologic examination and relevant investigation to determine the mechanism of stroke and also the risk factors. Patient’s neurological status was assessed via modified Rankin Scale (mRS) and National Institute of Health and Stroke Scale (NIHSS) which were done on admission and discharge. All patients had transcranial Doppler monitoring (DWL multidop × 2, Germany) for 1 h for the intracranial vessels to detect microembolic signals and MRI, MRA, and MRI perfusion (Seimens 1.5 T Espree, Germany) image to detect hypoperfusion signs.

### Statistical analysis

Analysis was performed using Statistical Package for Social Science (SPSS 15.0.1 for windows; SPSS Inc., Chicago, IL, 2001 (SPSS 15, Chicago, IL)). Mean and standard deviation (± SD) were used for parametric numerical data, while median and interquartile range for non-parametric numerical data. Chi-square test was used to examine the relationship between two qualitative variables. Fisher’s exact test was used to examine the relationship between two qualitative variables when the expected *N* is less than 5 in more than 20% of cells and kappa statistics to compute the measure of agreement between two investigational methods, kappas 0–0.20 are slight, 0.21–0.40 are fair, 0.41–0.60 are moderate, 0.61–0.80 are substantial (large), and 0.81–1 are almost perfect agreement. The equation used for sensitivity of diagnostic measures was true positive by the test/(true positive by the test + false negative by the test), and for specificity, the equation was true negative by the test/(true negative by the test + false positive by the test). PPV equals true positive by test/all positive by the test (true positive by the test + false positive by the test). NPV equals true negative by test/all negative by the test (true negative by the test + false negative by the test). Accuracy equals true negative by test + (true positive by test/total number). *P* < 0.05 was considered to be statistically significant for all tests.

## Results

**Fig. 1:**
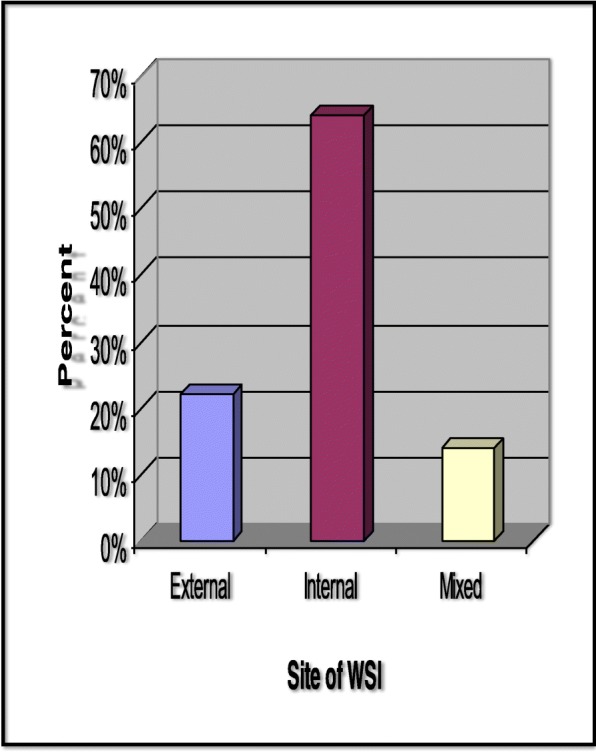
Site of watershed infarction among cases. Among those 50 patients with acute WSI, 11 watershed infarction (WSI) (22%) were located externally (cortical), while 32 WSI (64%) were located internally (sub cortical) and seven WSI (14%) were mixed, i.e., both external and internal. WSI watershed infarction

Those 50 patients with acute watershed infarction (WSI) included 38 males (76%) and 12 females (24%), and their age ranged from 37 to 81 years with mean age of 62.78 ± 10.26. Among those 50 patients with acute WSI, 11 WSI (22%) were located externally (cortical) while 32 WSI (64%) were located internally (sub cortical) and seven WSI (14%) were mixed, i.e., both external and internal (Fig. 1).

Forty-two patients (84%) showed hypoperfusion signs by MRI perfusion while 16 patients (32%) showed positive microembolic signals by TCD. Among those patients, eight WSI (16%) were due to embolic causes (i.e., positive microembolic signals by TCD and no hypoperfusion signs by MRI perfusion images), while 34 WSI (68%) were due to hypoperfusion causes (i.e., negative microembolic signals by TCD and positive hypoperfusion signs by MRI perfusion images). And eight WSI (16%) were mixed (Table 2).

**Table 2:** Description of watershed infarction characteristics (site and side), MRI perfusion, and transcranial Doppler results among cases

		*N*	Percentage
Site of watershed infarction by MRI	External	11	22.0
	Internal	32	64.0
	Mixed	7	14.0
Side of watershed infarction	Left	34	68.0
	Right	16	32.0
MRI perfusion	Hypoperfusion	42	84.0
	Normal	8	16.0
Microembolic signal by transcranial Doppler	Yes	16	32.0
	No	34	68.0
Etiology	Embolic only	8	16.0
	Hypo perfusion only	34	68.0
	Mixed	8	16.0

*MRI* magnetic resonance imaging, *N* number

Among the 42 patients with hypoperfusion signs, 22 patients (52.4%) had carotid plaque in the cervical ICA. These results indicate that patients who had carotid plaques are more liable to develop hypoperfusion WSI.

Among 16 patients that showed positive microembolic signals by TCD, 12 (75.1%) had impaired cardiac wall contractility. These results are significant with *P* value 0.001 indicating that patients with impaired cardiac wall contractility showed positive microembolic signals and it can be considered one of the embolic causes of WSI.

**Fig. 2:**
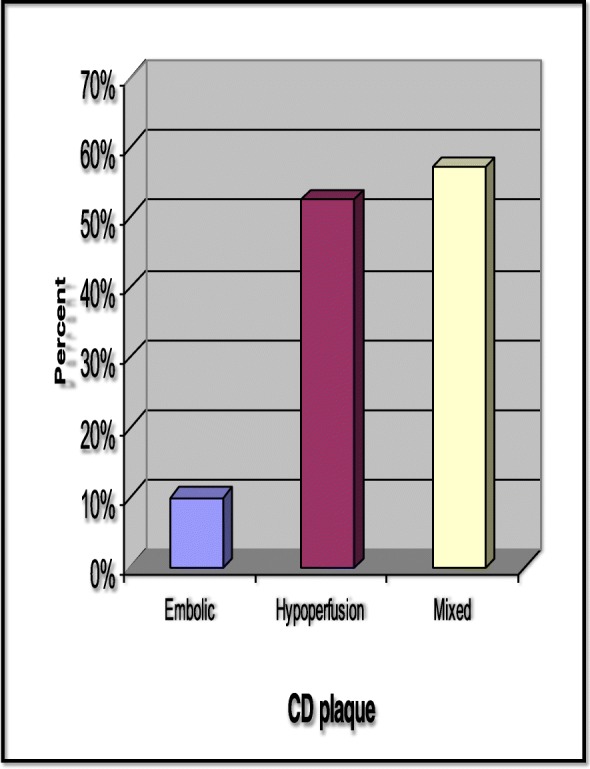
Carotid duplex plaques. Among the 23 patients who had plaques by carotid duplex in their internal carotid artery, only one of them developed embolic WSI, while 16 patients developed hypoperfusion WSI and six patients developed mixed WSI. CD carotid duplex

According to the results of cardiac wall contractility shown by TTE, one (12.5%) out of eight embolic WSI had normal wall contractility, while seven (87.5%) embolic WSI had impaired cardiac wall contractility (six patients with global hypokinesia and one patient with segmental hypokinesia). Twenty-eight (82.4%) out of 34 hypoperfusion WSI had normal wall contractility, and only six hypoperfusion WSI (17.6%) had impaired cardiac wall contractility (five patients with global hypokinesia and one patient with segmental hypokinesia). The result is highly significant with *P* value 0.008, and this indicates that patients who have impaired cardiac wall contractility more commonly develop embolic WSI. According to the results of presence of thrombus or not inside the heart by TEE, all hypoperfusion WSI (100%) had no thrombus inside the heart, while the six patients who had cardiac thrombus developed embolic or mixed WSI. These results are highly significant with *P* value 0.001 indicating that patients who have thrombus inside the heart are more liable to develop embolic or mixed WSI rather than hypoperfusion WSI. Among the 23 patients who had plaques by carotid duplex in their internal carotid artery, only one of them developed embolic WSI, while 16 patients developed hypoperfusion WSI and six patients developed mixed WSI. These results indicate that patients with carotid duplex plaques are more common to develop hypoperfusion or mixed WSI (Fig. 2, Table 3).

**Table 3:** Association between embolic, hypoperfusion, and mixed watershed infarction (by MRI perfusion and transcranial Doppler) and patients’ risk factors (chi-square test*)*

		Etiology						*P*	Significance
		Embolic only		Hypoperfusion only		Mixed			
		*N*	%	*N*	%	*N*	%		
Stroke/TIA	Yes	0	0.0	11	32.4	1	12.5	0.112	Non-significant
	No	8	100.0	23	67.6	7	87.5		
Wall contractility by transthoracic echo	Normal	1	12.5	28	82.4	3	37.5	0.008	Highly significant
	Segmental hypokinesia	1	12.5	1	2.9	2	25.0		
	Global hypokinesia	6	75.0	5	14.7	3	37.5		
Thrombus detected by TEE	Yes	4	50.0	0	0.0	2	25.0	0.001	Highly significant
	No	4	50.0	34	100.0	6	75.0		
Plaque	Yes	1	12.5	16	47.1	6	75.0	0.057	Non-significant
	No	7	87.5	18	52.9	2	25.0		
Plaque type by carotid duplex	Stable	0	0.0	8	50.0	3	50.0	1	Non-significant
	Unstable	1	100.0	8	50.0	3	50.0		
≥ 50% stenosis/occlusion	Yes	1	12.5	7	20.6	5	62.5	0.053	Non-significant
	No	7	87.5	27	79.4	3	37.5		

*TIA* transient ischemic attack, *TEE* trans-esophageal echocardiography

In a further analysis as regards the stability of these plaques and the etiology of WSI, eight out of 11 patients who had stable carotid plaque developed hypoperfusion WSI, while the 12 patients who had unstable carotid plaques developed either embolic, hypoperfusion, or mixed WSI. Although these results are not significant (*P* value 1), we can conclude that patients with stable plaques are more common to develop hypoperfusion WSI, while patients with unstable plaques are more common to develop any type of WSI.

As regards wall contractility by TTE, six out of 11 patients with external WSI had impaired cardiac wall contractility and five out of seven patients with mixed WSI had also impaired cardiac wall contractility, while 25 out of 32 patients with internal WSI had normal wall contractility. These results are significant with *P* value 0.033 indicating that patients with impaired cardiac wall contractility usually developed external WSI, while patients with internal WSI usually have normal wall contractility.

Among those 39 internal WSI that appeared by MRI of the brain, MRI perfusion detects hypoperfusion signs in 35 patients (89.7%). This indicates that patients with positive hypoperfusion signs by MRI perfusion most probably have internal WSI, as it is mostly due to hypoperfusion causes, reaching sensitivity 89.7% and accuracy 82%. These results are highly significant with *P* value < 0.001 (Table 4).

**Table 4:** Agreement between MRI of the brain and MRI perfusion as regards diagnosis of watershed infarction site (internal and mixed)

		Internal by MRI				Kappa	*P*	Sig
		Yes		No				
		*N*	%	*N*	%			
MRI perfusion	Hypoperfusion	37	94.9	5	45.5	0.548	˂ 0.001	HS
	Normal	2	5.1	6	54.5			

*MRI* magnetic resonance imaging, *N* number, *Sig* significance, *HS* highly significant

Among those 18 external WSI that appeared by MRI of the brain, TCD detects microembolic signals in 15 patients (83.3%). This indicates that patients with positive microembolic signals by TCD most probably have external WSI, as it is mostly due to embolic causes, reaching sensitivity 83.3% and accuracy 62% (Table 5). These results are significant with *P* value 0.001.

**Table 5:** Agreement between MRI of the brain and transcranial Doppler as regards diagnosis of watershed infarction site (external and mixed)

		External by MRI				Kappa	*P* value	Sig
		Yes		No				
		*N*	%	*N*	%			
Microembolic signal by TCD	Yes	11	61.1	5	15.6	0.466	0.001	HS
	No	7	38.9	27	84.4			

*MRI* magnetic resonance imaging, *Sig* significance, *HS* highly significant, *TCD* transcranial Doppler

## Discussion

Hemodynamic failure and microembolization were proposed to explain the phenomenon of watershed infarction in patients with acute ischemic stroke [12]. In this study, we aimed to differentiate between hypoperfusion and microemboli as etiology of acute ischemic stroke in watershed zone. We included 50 patients with acute WSI which included 38 males (76%) and 12 females (24%), and their age ranged from 37 to 81 years with mean age of 62.78 ± 10.26. This wide range of age group were included in this study to give chance for inclusion of embolic etiologies which most probably occur in young age group (especially of cardiac causes) and also hypoperfusion etiologies that mostly occur in older age group. Male predominance in our study explains that acute stroke (especially with specific etiologies as microemboli and hypoperfusion due to extracranial carotid advanced atherosclerotic diseases) occurs more commonly in males [4]. Among those 50 patients, eight patients (16%) had WSI due to embolic causes only. Thirty-four patients (68%) had WSI due to hypoperfusion causes only, and eight patients (16%) had WSI due to mixed causes. We involve both cardiac patients and patients with carotid artery plaques or stenosis and occlusion to detect both embolic and hypoperfusion causes of WSI. We detect embolic causes of WSI by using TCD with 61.1% sensitivity, 84.4% specificity, and 76% accuracy. And we detect the hypoperfusion causes of WSI by using MRI perfusion studies to detect hypoperfusion signs with 94.9% sensitivity, 54.5% specificity, and 86% accuracy. Although there are relative low sensitivity of TCD and specificity of MRI perfusion, according to our study, those methods have good accuracy in detection of microembolic and hypoperfusion etiologies and can be used in clinical practice. As regards the embolic causes, among the 50 patients included in our study, 16 patients (32%) showed microembolic signals by TCD. Those 16 patients were characterized by the following: 12 patients out of 16 had impaired cardiac wall contractility either segmental or global hypokinesia as detected by TTE (with significant result *P* value.001), six patients out of 16 had cardiac thrombus as detected by TEE (with significant result *P* value.001), and seven patients out of 16 had carotid plaques and four of these carotid plaques were unstable and ulcerative as detected by carotid duplex (this result is not significant, and this can be explained by low number of cases). So we can conclude that the embolic causes of WSI are impaired cardiac wall contractility, presence of cardiac thrombus, and presence of unstable carotid plaques. In our study, among 34 patients who had hypoperfusion WSI, 28 (82.4%) had normal wall contractility, while 12 patients out of 18 patients who had impaired cardiac wall contractility, whether segmental or global hypokinesia, developed embolic and mixed WSI. In a previous study done by Martin [8], the contribution of microembolic signal detection in cardio embolic stroke stated that there is a high prevalence of microembolic signal in patients with cardiac embolism that even topped the prevalence found in patients with symptomatic carotid stenosis. However, in his study, the results were not significant and he attributed that to the low number of cases who participated in this study and stated that larger studies would be needed to strengthen this role. Also, he did not involve patients with carotid disease as a source of microembolism. Another study done by Ramez and his colleagues [11] supported the idea that in symptomatic ICA disease, the deep WSI may result from hemodynamic instability through severe lumen stenosis but also from microemboli through plaque inflammation. And this was consistent with our results. Another study done by Kimura and his coworkers [6] used the TCD to detect the microembolic signals in patients with acute ischemic stroke and they stated that TCD can provide early clues to the mechanisms of stroke during the acute phase and they stated that the main sources of microemboli in ICA stenosis are ulceration and thrombus formation of the atheromatous plaque. Also, they stated that small emboli may occlude peripheral brain arteries and generate spotty cortical or sub cortical infarcts and that microembolic signals may indicate the presence of unstable plaques or platelet aggregation in arterial disease that promote artery to artery embolism. However, they did not use the TCD in cardiac patients to detect heart as a source of embolic causes of WSI [6].

According to the thrombus detected by TEE, six patients (100%) who had cardiac thrombus developed embolic and mixed WSI and no patient with hypoperfusion WSI was having cardiac thrombus.

According to the carotid duplex plaques, eight patients (50%), with stable carotid plaques, out of 16 patients who had carotid plaques developed hypoperfusion WSI, while the 12 patients with unstable ulcerative plaques developed embolic, hypoperfusion, or mixed WSI.

The previous results were significant to highly significant in the cardiac causes but not significant in the carotid plaque causes, and this can be explained by the low number of the cases. But in general, we can conclude that the causes that cause embolic WSI (or the embolic causes) are impaired cardiac wall contractility or presence of cardiac thrombus or presence of unstable ulcerative plaque, while the causes that cause hypoperfusion WSI (or hypoperfusion causes) are presence of carotid plaques [11].

There are no sufficient data to associate between the causes of WSI and its type whether external, internal, or mixed. In our study, we correlate between the causes of WSI and its site by MRI brain images. In our study, 25 patients (78%) out of 32 patients who had normal wall contractility developed internal WSI only, while 11 patients (61%) out of 18 patients who had impaired cardiac wall contractility developed external and mixed WSI.

## Conclusion

We detected the etiology of WSI, either embolic by using TCD or hypoperfusion by using MRI perfusion. The embolic causes of WSI (impaired cardiac wall contractility or presence of cardiac thrombus or presence of unstable ulcerative plaque) usually cause external or mixed WSI, while the hypoperfusion causes of WSI (stable carotid plaques) usually cause internal WSI.

## Abbreviations

ACA: Anterior cerebral artery
CWS: Cortical watershed strokes
DM: Diabetes mellitus
HTN: Hypertension
ICA: Internal carotid artery
IWS: Internal watershed strokes
IWS: Internal watershed strokes
MCA: Middle cerebral artery
MRI: Magnetic resonance imaging
mRS: Modified Rankin Scale
NIHSS: National Institute of Health and Stroke Scale
PCA: Posterior cerebral artery
SD: Standard deviation
SPSS: Statistical Package for Social Science
TCD: Transcranial Doppler
TEE: Trans-esophageal echocardiography
TIA: Transient ischemic attack
TTE: Transthoracic echocardiography
WSI: Watershed infarction
